# CYP1A1 Enzymatic Activity Influences Skin Inflammation Via Regulation of the AHR Pathway

**DOI:** 10.1016/j.jid.2020.11.024

**Published:** 2021-06

**Authors:** Mariela Kyoreva, Ying Li, Mariyah Hoosenally, Jonathan Hardman-Smart, Kirsten Morrison, Isabella Tosi, Mauro Tolaini, Guillermo Barinaga, Brigitta Stockinger, Ulrich Mrowietz, Frank O. Nestle, Catherine H. Smith, Jonathan N. Barker, Paola Di Meglio

**Affiliations:** 1 AhRimmunity Lab, The Francis Crick Institute, London, United Kingdom; 2 St John’s Institute of Dermatology, King’s College London, London, United Kingdom; 3 Psoriasis Centre at the Department of Dermatology, University Medical Centre Schleswig-Holstein, Kiel, Germany

**Keywords:** α-NF, α-naphthoflavone, EROD, ethoxyresorufin-O-deethylase, FICZ, 6-formylindolo[3,2-b]carbazole, K, keratin, KC, keratinocyte, PAH, polycyclic aromatic hydrocarbon, Th17, T helper type 17

## Abstract

The AHR is an environmental sensor and transcription factor activated by a variety of man-made and natural ligands, which has recently emerged as a critical regulator of homeostasis at barrier organs such as the skin. Activation of the AHR pathway downmodulates skin inflammatory responses in animal models and psoriasis clinical samples. In this study, we identify CYP1A1 enzymatic activity as a critical regulator of beneficial AHR signaling in the context of skin inflammation. Mice constitutively expressing *Cyp1a1* displayed increased CYP1A1 enzymatic activity in the skin, which resulted in exacerbated immune cell activation and skin pathology, mirroring that observed in *Ahr-deficient* mice. Inhibition of CYP1A1 enzymatic activity ameliorated the skin immunopathology by restoring beneficial AHR signaling. Importantly, patients with psoriasis displayed reduced activation of the AHR pathway and increased CYP1A1 enzymatic activity compared with healthy donors, suggesting that dysregulation of the AHR/CYP1A1 axis may play a role in inflammatory skin disease. Thus, modulation of CYP1A1 activity may represent a promising alternative strategy to harness the anti-inflammatory effect exerted by activation of the AHR pathway in the skin.

## Graphical abstract

**Figure:**
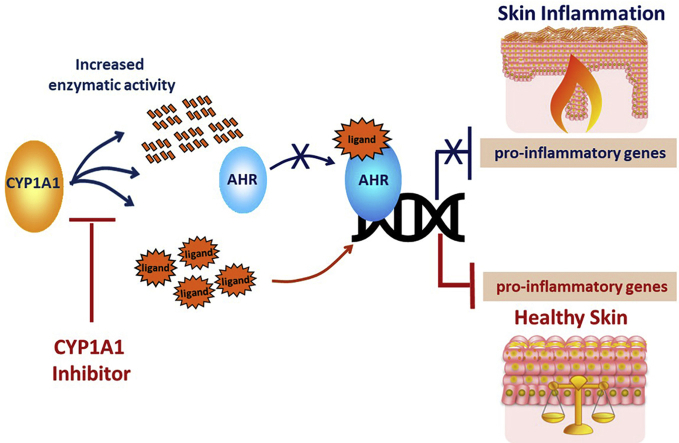

## Introduction

The environmental sensor AHR is an evolutionarily conserved transcription factor activated by either xenobiotic, such as dioxins and polycyclic aromatic hydrocarbons (PAHs), or physiological ligands, that is, indole compound derivatives of tryptophan of dietary, microbial, or host metabolism origin.

Well-known for its detrimental role in mediating xenobiotic-driven cellular toxicity of healthy tissue (Haarmann-Stemmann et al., 2015), AHR is increasingly emerging as a critical positive regulator of cellular homeostasis during inflammation, especially at barrier organs such as the skin and the gut (Esser and Rannug, 2015; Stockinger et al., 2014). Activation of AHR signaling controls inflammation and autoimmunity through direct and indirect transcriptional regulation of proinflammatory genes and maintenance of barrier integrity (Rothhammer and Quintana, 2019).

AHR is expressed in the skin and plays a beneficial and anti-inflammatory role in both autoimmune and allergic skin inflammation (Di Meglio et al., 2014a; van den Bogaard et al., 2013).

Psoriasis is an IL-17–driven, chronic inflammatory skin disease resulting from the combination of genetic susceptibility, environmental triggers, and dysregulated immune responses (Di Meglio et al., 2014b; Prinz et al., 2020). We have previously shown that lack of AHR signaling exacerbates the severity of psoriasiform inflammation in mice by unleashing excessive production of chemokines and cytokines in response to proinflammatory stimuli (Di Meglio et al., 2014a). Conversely, AHR ligation by the tryptophan-derived ligand 6-formylindolo[3,2-b]carbazole (FICZ) ameliorated the transcriptional profile of ex vivo skin biopsies from patients with psoriasis and reduced the severity of psoriasiform inflammation in mice.

Therefore, the AHR pathway is an attractive target for the control of inflammatory skin disease, and topical application of tapinarof, a naturally derived AHR ligand (Smith et al., 2017), has shown promising efficacy in phase II clinical trials for psoriasis and atopic dermatitis (Peppers et al., 2019; Robbins et al., 2019).

Central to the regulation of the AHR pathway is the induction of CYP1 enzymes, particularly CYP1A1. Besides its role in biotransformation and detoxification of xenobiotics, CYP1A1 is involved in the degradation of physiological AHR ligands, thereby providing an autoregulatory feedback mechanism that terminates AHR signaling and prevents the persistent activation and overt toxicity induced by xenobiotics (Effner et al., 2017; Stockinger et al., 2014; Wincent et al., 2012). In this study, we hypothesize that CYP1A1 feedback regulates skin inflammation and that upregulation of its enzymatic activity may occur in patients with psoriasis, disrupting the anti-inflammatory effect mediated by physiological AHR signaling. Thus, inhibition of CYP1A1 activity may represent an alternative strategy to ameliorate skin inflammation through AHR.

## Results

### The AHR-CYP1A1 pathway in psoriasiform skin inflammation in mice

**Figure 1:**
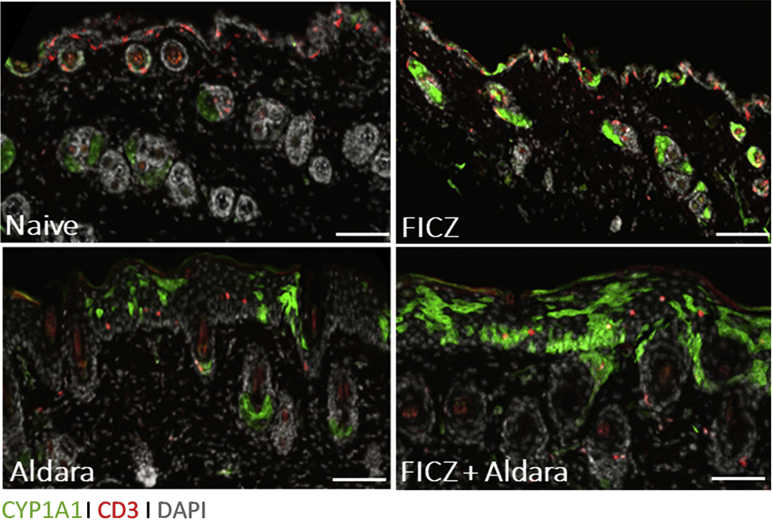
**CYP1A1 expression in naive murine skin and during skin inflammation**. Skin section of *Cyp1a1*^*Cre*^ × *R26R*^*eYFP*^ reporter mice, either naive or treated with FICZ for 7 days or with Aldara for 5 days in the presence or absence of FICZ, stained for CD3 (red), CYP1A1 (green), and DAPI (gray). Bar = 100 μm. Images are representative of two independent experiments, n = 2 and 3 mice per group. FICZ, 6-formylindolo[3,2-b]carbazole.

**Supplementary Figure S1:**
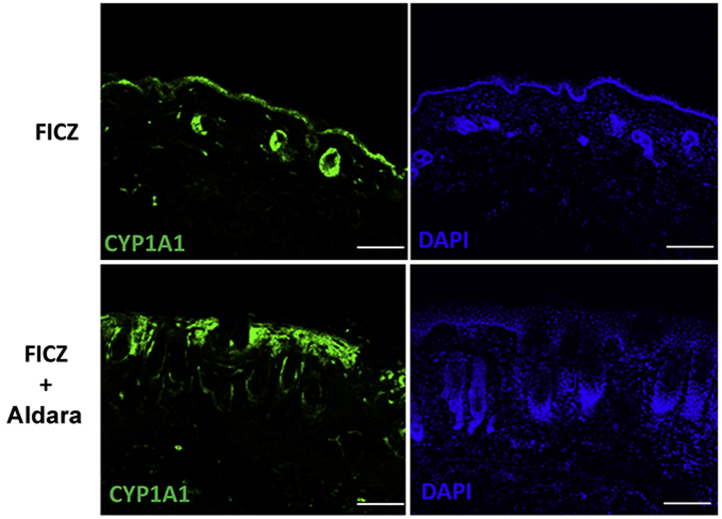
**CYP1A1 expression in naive murine skin and during skin inflammation.** Skin section of *Cyp1a1*^*Cre*^ × *R26R*^*eYFP*^ reporter mice treated topically with FICZ (5 μg in 50 μl corn oil per mouse) for 6 days or with FICZ plus Aldara in the last 5 days, stained for CYP1A1 (green) and DAPI (blue). Bar = 100 μm. Images are representative of three mice per group. FICZ, 6-formylindolo[3,2-b]carbazole.

To study the activation of the AHR pathway during the Aldara-induced psoriasiform skin inflammation model, we made use of the *Cyp1a1*^*Cre*^ × *R26R*^*eYFP*^ reporter mouse strain that reports AHR activity and CYP1A1 protein expression through the induction of the eYFP (Schiering et al., 2017). CYP1a1 expression was limited but detectable in epidermal keratinocytes (KCs) and sebaceous glands of naive reporter mice (Figure 1), in keeping with the transient AHR signaling induced by physiological ligands. As expected, systemic administration of FICZ for 7 days intraperitoneally induced a noticeable activation of the AHR pathway in the skin. Interestingly, topical application of Aldara cream for 5 days also markedly upregulated AHR signaling, which was further enhanced by concomitant FICZ administration (Figure 1). Similar results were obtained when FICZ was administered topically (Supplementary Figure S1a). Thus, in keeping with its anti-inflammatory effect (Di Meglio et al., 2014a), the AHR pathway is activated during psoriasiform skin inflammation, and this is further enhanced by exogenous supplementation with a physiological ligand.

### Characterization of the skin of *R26*^*Cyp1a1*^ mouse

**Figure 2:**
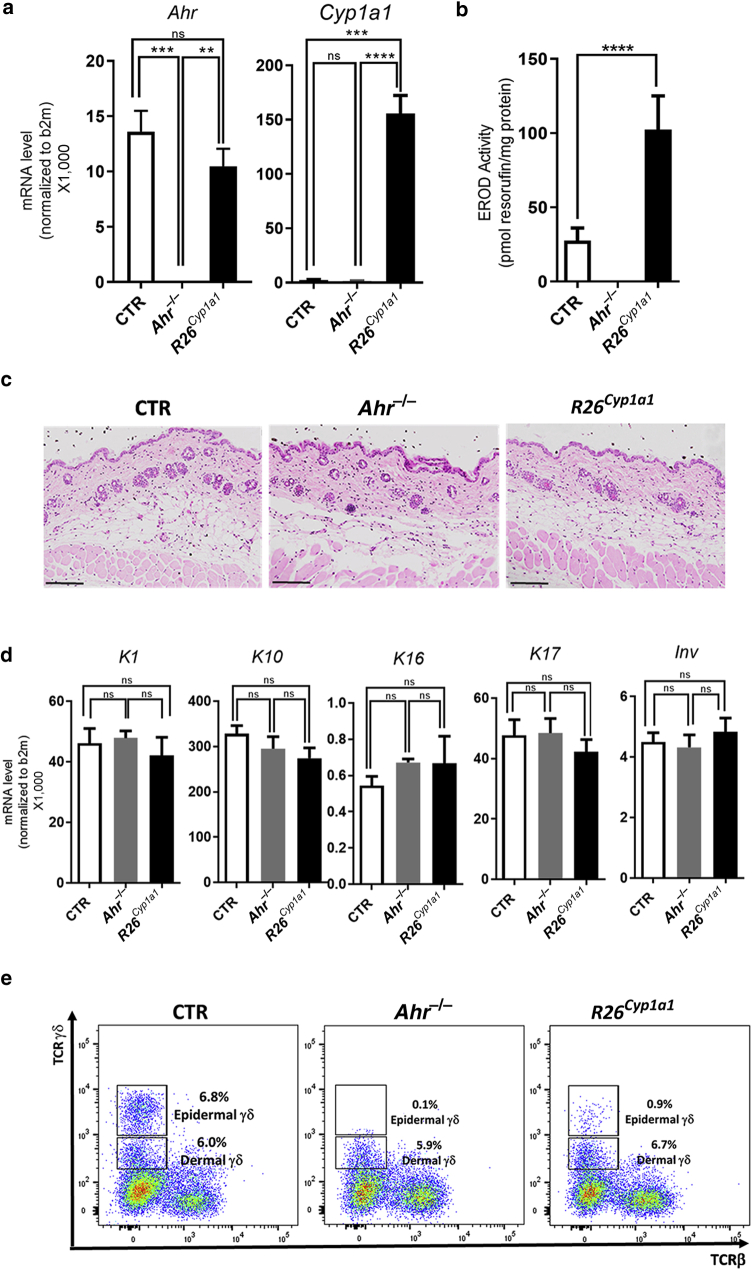
**Characterization of *R26*^*Cyp1a1*^ mouse strain.** (**a**) *AhR* and *Cyp1a1* expression in the skin of naive CTR, *AhR*^*−/−*^, and *R26*^*Cyp1a1*^ mice. (**b**) CYP1A1 enzymatic activity in tail skin of naive CTR, *AhR*^*−/−*^, and *R26*^*Cyp1a1*^ mice. (**c**) Representative images of skin histological sections of naive CTR, *AhR*^*−/−*^, and *R26*^*Cyp1a1*^ mice. (**d**) mRNA expression of skin differentiation and hyperproliferation genes in whole skin of naive CTR, *AhR*^*−/−*^, and *R26*^*Cyp1a1*^ mice. (**e**) Representative flow cytometry dot plots showing the expression of TCRβ and TCRγδ in naive CTR, *AhR*^*−/−*^, and *R26*^*Cyp1a1*^ mice. Graphs show mean ± SEM of pooled results of at least two independent experiments (n = 3–6 per group). ∗∗*P* < 0.01, ∗∗∗*P* < 0.001, and ∗∗∗∗*P* < 0.0001. CTR, control; EROD, ethoxyresorufin-O-deethylase; K, keratin; ns, nonsignificant.

**Supplementary Figure S2:**
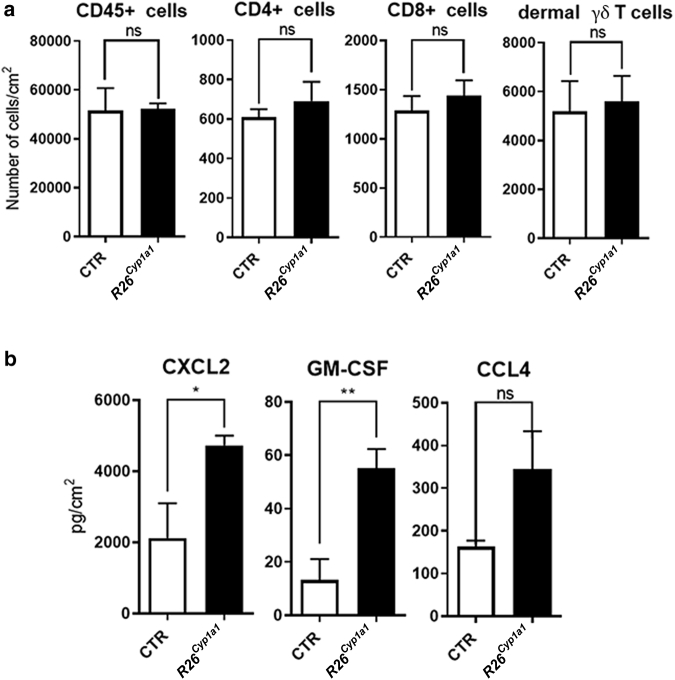
**Characterization of *R26*^*Cyp1a1*^ mouse strain.** (**a**) Numbers of total CD45+ immune cells and T-cell population in the skin of naive CTR and *R26*^*Cyp1a1*^ mice analyzed by flow cytometry. (**b**) Proinflammatory proteins in the whole skin from Aldara-treated CTR and *R26*^*Cyp1a1*^ mice. Graphs show mean ± SEM of pooled results of (**a**) two independent experiments or (**b**) of one representative of two independent experiments (n = 3–6 mice per group). ∗*P* < 0.05, ∗∗*P* < 0.01. CTR, control; ns, nonsignificant.

Next, we sought to evaluate the role of CYP1A1 negative feedback in the control of skin homeostasis. To this end, we used the *R26*^*Cyp1a1*^ mouse strain, in which *Cyp1a1* is ubiquitously expressed under the control of the ROSA26 promoter (Schiering et al., 2017). First, we characterized the skin of naive adult mice in the absence of any provoked inflammation. We detected a significantly higher expression of *Cyp1a1* but not *Ahr* mRNA in the skin of *R26*^*Cyp1a1*^ mice compared with that of wild-type control mice (Figure 2a). Next, we measured CYP1A1 enzymatic activity in epidermal sheet biopsies obtained from tail skin. Ethoxyresorufin-O-deethylase (EROD) activity was significantly higher in *R26*^*Cyp1a1*^ mice than in control mice (Figure 2b). As expected, no *Cyp1a1* or *Ahr* mRNA nor EROD activity were detected in the skin of *Ahr*^*−/−*^ mice. Histological analysis of back skin full-thickness biopsies showed a similar, normal skin pattern in all the three strains analyzed, with the skin of *R26*^*Cyp1a1*^ comparable with that of the control and *Ahr*^*-/-*^ mice (Figure 2c). Similarly, mRNA of early (keratin (K) 1 and K10 mRNAs, *K1* and *K10)* and late *(Inv)* skin differentiation and hyperproliferation genes (K16 and K17 mRNAs, *K16* and *K17*) did not differ (Figure 2d). Total CD45+ immune cells and major T-cell populations in the skin did not vary (Supplementary Figure S2a), with one notable exception, because the nearly total loss of epidermal γδ T cells previously reported in *Ahr*^*−/−*^ mice (Kadow et al., 2011; Li et al., 2011) was mirrored by a profound reduction in the *R26*^*Cyp1a1*^ mice (Figure 2e).

Taken together, *R26*^*Cyp1a1*^ mice displayed a high level of *Cyp1a1* mRNA and increased enzymatic activity in the skin; yet, their skin was indistinguishable from that of control mice but for the significant reduction of epidermal γδ T cells.

### Constitutive expression of *Cyp1a1* exacerbates psoriasis-like skin inflammation phenocopying AHR deficiency

Next, we used the Aldara-induced psoriasiform skin inflammation model to evaluate the effect of *Cyp1a1* overexpression in the context of skin pathology compared with control or *Ahr*^*−/−*^ mice.

**Figure 3:**
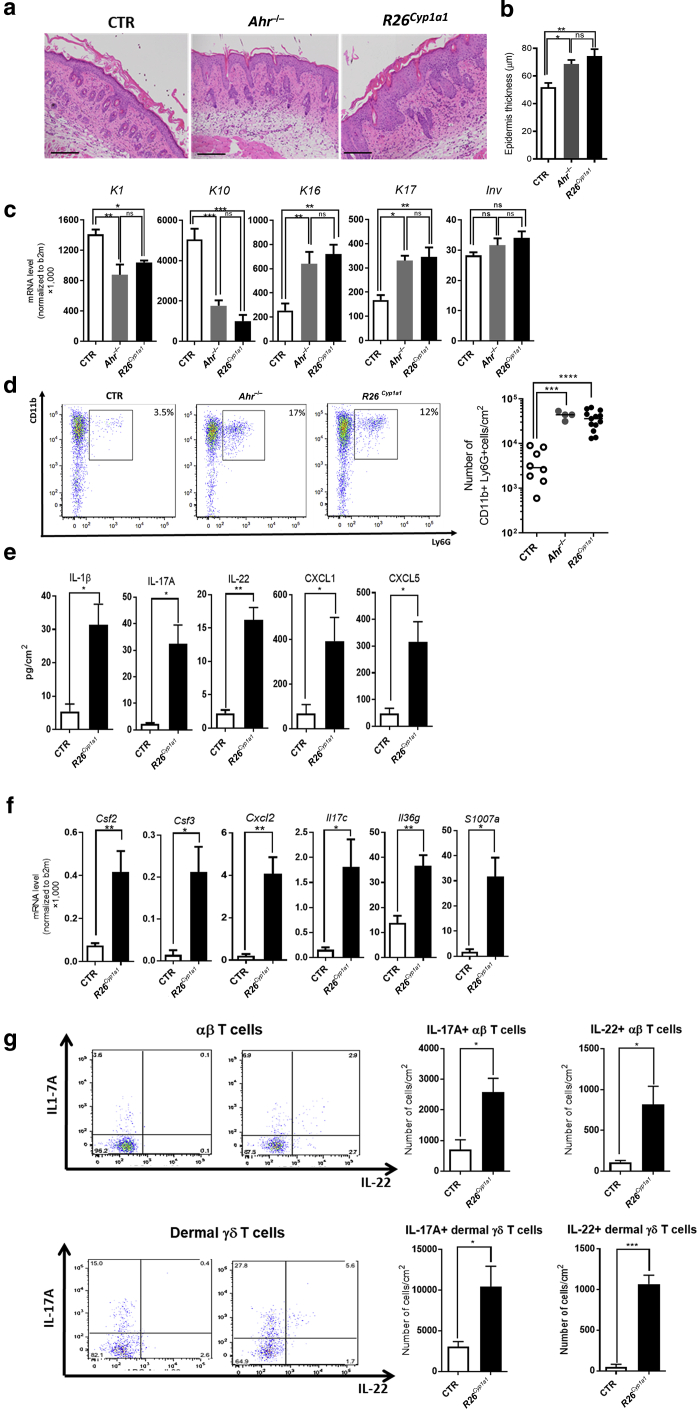
**Constitutive expression of *Cyp1a1* phenocopies *Ahr* deficiency and exacerbates skin pathology.** (**a**) Representative images of skin histological sections from Aldara-treated CTR, *AhR*^*−/−*^, and *R26*^*Cyp1a1*^ mice on day 6 (bars = 100 μm). (**b**) Quantification of the epidermal thickness of CTR, *AhR*^*−/−*^, and *R26*^*Cyp1a1*^ mice. (**c**) mRNA expression of skin differentiation and hyperproliferation genes in whole skin from Aldara-treated CTR, *AhR*^*−/−*^, and *R26*^*Cyp1a1*^ mice. (**d**) Representative flow cytometry dot plots showing frequency (left) and scatter plot graph (right) showing the numbers of neutrophils in naive CTR, *AhR*^*−/−*^, and *R26*^*Cyp1a1*^ mice. (**e**) Proinflammatory proteins in the whole skin from Aldara-treated CTR and *R26*^*Cyp1a1*^ mice. (**f**) mRNA expression of proinflammatory mediators in whole skin from Aldara-treated CTR and *R26*^*Cyp1a1*^ mice. (**g**) Representative flow cytometry dot plots (left) and bar plot graphs (right) showing the numbers of αβ (top) and γδ (bottom) T cells expressing IL-17 and IL-22 in the skin of Aldara-treated CTR and *R26*^*Cyp1a1*^ mice. Graphs show (**b, c, e, f, g**) mean ± SEM and (**d**) individual values of (**e, g**) one representative experiment or (**b–d**) pooled results of two to three independent experiments (n = 3–13 mice per group). ∗*P* < 0.05, ∗∗*P* < 0.01, ∗∗∗*P* < 0.001, and ∗∗∗∗ *P* < 0.0001. CTR, control; K, keratin; ns, nonsignificant.

As previously reported (Di Meglio et al., 2014a), topical application of Aldara cream for 5 days induced exacerbated psoriasiform phenotype in *Ahr*^*−/−*^ mice compared with that in the control mice. This was characterized by increased acanthosis (Figure 3a and b), decreased KC early differentiation and increased hyperproliferation (Figure 3c), and increased skin neutrophilia (Figure 3d) in the skin of Aldara-treated *Ahr*^*−/−*^ and *R26*^*Cyp1a1*^ mice. Molecular analysis of the skin of *R26*^*Cyp1a1*^ mice revealed increased protein levels of IL-1β, IL-17A, IL-22, CXCL1, CXCL2, CXCL5, and GM-CSF (Figure 3e and Supplementary Figure S2b) as well as increased mRNA expression for *Csf2, Csf3*, *Il17c*, *Il36g,* and *S100a7a* (Figure 3f)*.* Finally, the number of αβ+ and γδ+ T cells producing IL-17A and IL-22 was increased (Figure 3g).

Taken together, the exacerbated skin phenotype induced by Aldara treatment in *R26*^*Cyp1a1*^ mice phenocopies that of *Ahr*^*−/−*^ mice, suggesting that increased CYP1A1 enzymatic activity in *R26*^*Cyp1a1*^ mice is as detrimental as the lack of AHR signaling in *Ahr*^*−/−*^ mice.

### CYP1A1 inhibition ameliorates Aldara-induced psoriasiform inflammation in *R26*^*Cyp1a1*^ mice

**Figure 4:**
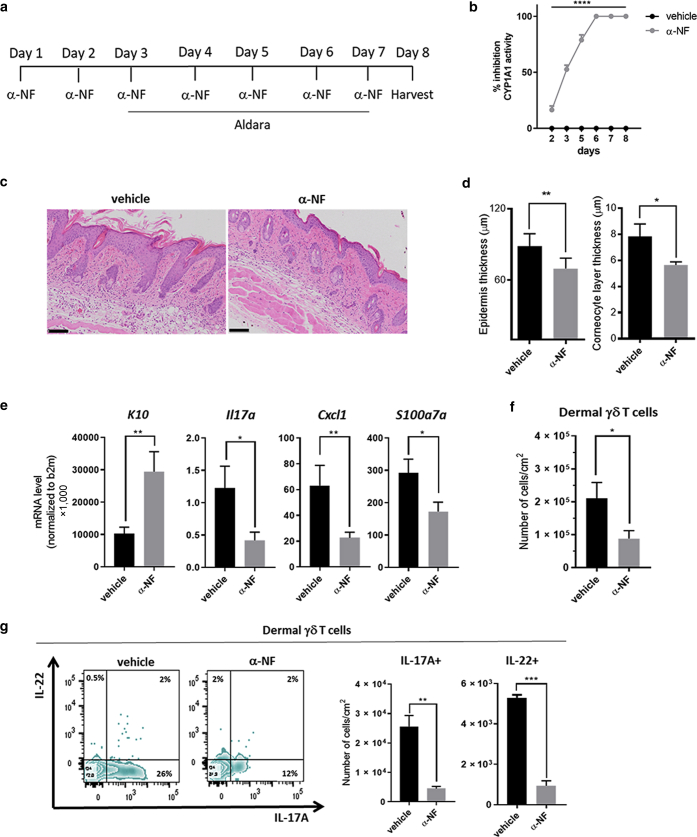
**CYP1A1 inhibition ameliorates psoriasiform inflammation in *R26*^*Cyp1a1*^ mice.** (**a**) Experimental design of CYP1A1 enzymatic activity blockade in *R26*^*Cyp1a1*^ mice treated with Aldara for 5 days. The control group received vehicle (corn oil) instead of α-NF. (**b**) Inhibition of CYP1A1 activity measured as EROD activity in tail epidermal sheets of *R26*^*Cyp1a1*^ mice receiving α-NF or vehicle control expressed as a percentage of enzymatic activity in untreated mice at different time points. (**c**) Representative images of skin histological sections from vehicle- or α-NF‒treated *R26*^*Cyp1a1*^ mice on day 8 of the experiment (bars = 100 μm). (**d**) Quantification of epidermal (left) or corneocytes (right) thickness of vehicle- or α-NF‒treated *R26*^*Cyp1a1*^ mice on day 8 of the experiment. (**e)** mRNA expression of proinflammatory mediators and KC differentiation marker in whole skin from vehicle- or α-NF‒treated *R26*^*Cyp1a1*^ mice on day 8. (**f**) Numbers of dermal γδ T cells in vehicle- or α-NF‒treated *R26*^*Cyp1a1*^ mice on day 8. (**g**) Representative flow cytometry zebra plots showing frequency (left) and bar plot graphs (right) showing the numbers of dermal γδ T cells producing IL-17A and IL-22 in vehicle- or α-NF‒treated *R26*^*Cyp1a1*^ mice on day 8. Graphs show `(**b, g**) mean ± SEM of one representative or (**d–f**) pooled results of two to three independent experiments (n = 3–11 mice per group). ∗*P* < 0.05, ∗∗*P* < 0.01, ∗∗∗*P* < 0.001, and ∗∗∗∗ *P* < 0.0001. α-NF, α-naphthoflavone; EROD, ethoxyresorufin-O-deethylase; K, keratin; KC, keratinocyte.

To further evaluate the effect of aberrant CYP1A1 enzymatic activity during skin inflammation, we used the CYP1A1 inhibitor α-naphthoflavone (α-NF) (Wincent et al., 2012) to restore homeostatic CYP1A1 activity. α-NF, administered intraperitoneally daily starting 2 days before Aldara (Figure 4a), significantly inhibited CYP1A1 activity in the skin already after one administration, reaching 100% inhibition by day 6 (Figure 4b). Skin histological analysis on day 8 revealed a marked amelioration of Aldara-induced skin pathology in *R26*^*Cyp1a1*^ mice treated with α-NF compared with that in vehicle-treated mice (Figure 4c). Both epidermal acanthosis and corneocyte layer were significantly reduced in α-NF‒treated mice (Figure 4d), paralleled by significantly increased expression of K10 gene, *K10* (Figure 4e). Gene expression analysis of skin tissue also showed a significant reduction in *Il-17a*, *Cxcl1,* and *S100a7a* expression (Figure 4e). Moreover, the number of dermal γδ T cells infiltrating the skin of α-NF‒treated *R26*^*Cyp1a1*^ mice (Figure 4f) and producing IL-17A and IL-22 (Figure 4g) was decreased.

### The AHR-CYP1A1 pathway is dysregulated in psoriasis

Given the exacerbated skin inflammation induced by Aldara in the presence of increased CYP1A1 activity in *R26*^*Cyp1a1*^ mice, we sought to translate our findings in the context of human skin pathology using clinical samples (Supplementary Table S1).

**Figure 5:**
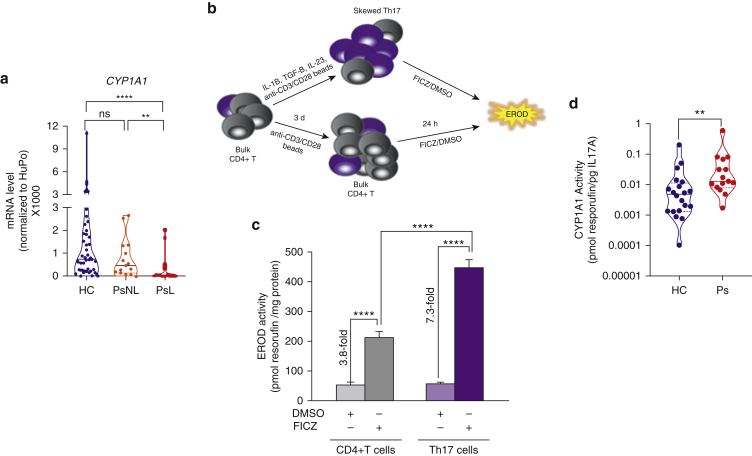
**The AHR/CYP1A1 pathway is dysregulated in Ps.** (**a**) mRNA expression of *CYP1A1* in whole skin biopsies from HCs NN skin (n = 42), PsNL skin (n = 15), and PsL skin (n = 22). (**b**) Schematic depicting the experimental design of generation of in vitro skewed Th17 cells from bulk CD4+ T cells and induction of CYP1A1 enzymatic activity measured as EROD activity. Bulk CD4+ T cells cultured in the absence of skewing cytokines were used as control. (**c**) CYP1A1 enzymatic activity induced by AHR ligand FICZ measured as EROD activity. DMSO was used as vehicle control in the in vitro skewed Th17 cells or control bulk CD4+ T cells. Data shown are expressed as mean ± SEM of one representative of at least three independent experiments, each run in quadruplicate. (**d**) CYP1A1 enzymatic activity in the in vitro skewed Th17 cells from HCs (n = 20) or patients with Ps (n = 15), measured as EROD activity normalized to the amount of IL-17A secreted in culture. Violin plot shows mean and individual values. ∗∗*P* < 0.01 and ∗∗∗∗*P* < 0.0001. d, day; EROD, ethoxyresorufin-O-deethylase; FICZ, 6-formylindolo[3,2-b]carbazole; h, hour; HC, healthy control; NN, normal; ns, nonsignificant; Ps, Psoriasis; PsL, psoriasis lesional; PsNL, psoriasis nonlesional; Th17, T helper type 17.

First, we evaluated the expression of CYP1A1 in the skin comparing normal (n = 42), lesional psoriasis (n = 22), and nonlesional psoriasis (n = 15) biopsy samples. *CYP1A1* expression did not differ in normal and nonlesional psoriasis biopsy samples but was significantly lower in lesional psoriasis than in nonlesional psoriasis and normal biopsy samples (Figure 5a). Thus, the expression of *CYP1A1* is significantly decreased in lesional skin, suggesting that activation of the AHR pathway is impaired in psoriasis.

**Supplementary Figure S3:**
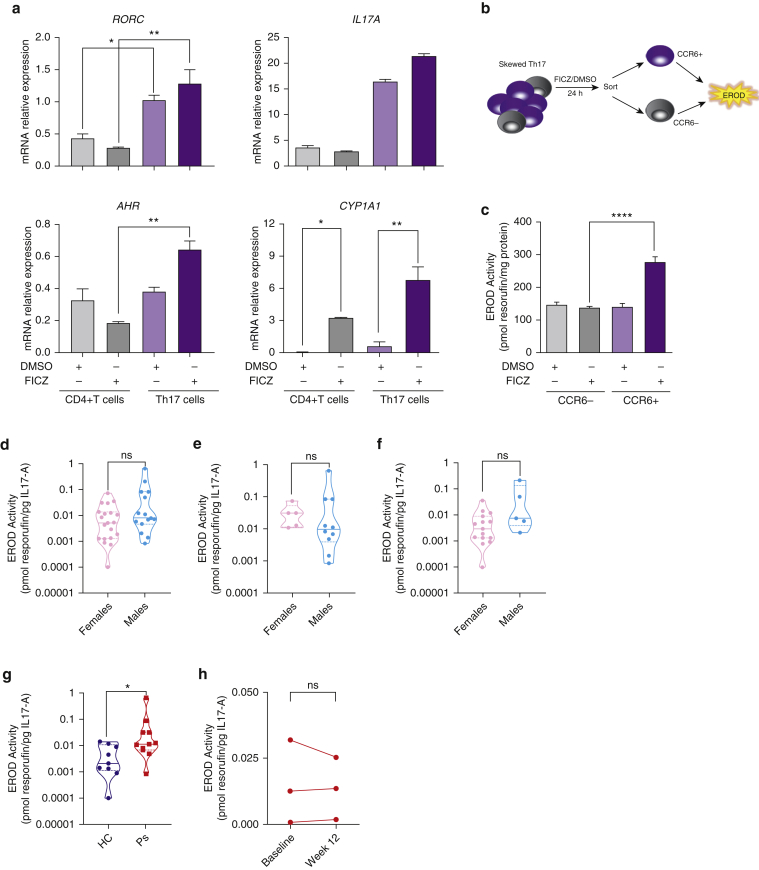
**The AHR/CYP1A1 pathway is dysregulated in Ps.** (**a**) mRNA expression of *RORC*, *IL17A*, *AHR,* and *CYP1A1* genes in the in vitro skewed Th17 cells or control bulk CD4+ T cells in the presence of FICZ or DMSO vehicle control on day 4 of the experiment. (**b**) Schematic depicting experimental design for measuring EROD activity in CCR6+ and CCR6− cells sorted from in vitro skewed Th17 cells. (**c**) CYP1A1 enzymatic activity induced by AHR ligand FICZ measured as EROD activity in sorted CCR6+ and CCR6− cells. DMSO was used as control. (**d‒f**) CYP1A1 enzymatic activity in the in vitro skewed Th17 cells (**d**) in the combined cohort or (**e**) in healthy controls or (**f**) in patients with Ps, measured as EROD activity normalized to the amount of IL-17A secreted in culture. (**g**) CYP1A1 enzymatic activity in the in vitro skewed Th17 cells from nonsmokers HCs (n = 9) or patients with Ps (n = 11), measured as EROD activity normalized to the amount of IL-17A secreted in culture. (**h**) CYP1A1 enzymatic activity in the in vitro skewed Th17 cells from three patients with Ps before commencing biological therapy (baseline) and at week 12, measured as EROD activity normalized to the amount of IL-17A secreted in culture. (**a, b**) Data shown are expressed as mean ± SEM of one representative of two independent experiments, each run in duplicate. (**c–e**) Violin plots show mean and individual values. ∗*P* < 0.05, ∗∗*P* < 0.01, and ∗∗∗∗*P* < 0.0001. EROD, ethoxyresorufin-O-deethylase; FICZ, 6-formylindolo[3,2-b]carbazole; h, hour; HC, healthy control; ns, nonsignificant; Ps, psoriasis; Th17, T helper type 17.

Next, we investigated CYP1A1 activity in healthy controls and in patients with psoriasis. To this end, we sought to establish a reliable and robust in vitro system to measure CYP1A1 activity in human primary blood cells. CYP1A1 activity can be measured in expanded human PBMCs stimulated with AHR agonist (Smart and Daly, 2000). However, because AHR expression may vary across different immune cell populations and immune cell frequency may vary between individuals (Roederer et al., 2015), total PBMCs may not be a reliable and robust model system. We screened several major immune cell populations, including B cells, monocytes, and macrophages, to identify the best model system (data not shown). In keeping with the high AHR expression in murine T helper type 17 (Th17) cells (Veldhoen et al., 2008), we found that in vitro skewed human Th17 cells provided a robust experimental system to measure CYP1A1 activity in healthy donors compared with bulk CD4+ T cells (Figure 5b). Next, we activated AHR and induced CYP1A1 expression by stimulating the cells with the AHR ligand FICZ or vehicle control (DMSO, Sigma-Aldrich, St. Louis, MO) for 24 hours before measuring CYP1A1 activity by EROD assay. Gene expression analysis confirmed successful differentiation and expansion of Th17 cells and activation of the AHR pathway by FICZ (Supplementary Figure S3a). Expression of *IL-17A* and *RORC* was higher in the in vitro skewed Th17 cells than in bulk CD4+ cells cultured in the absence of skewing cytokines. *AHR* expression was detected across all samples, with in vitro skewed Th17 cells expressing the highest levels. As expected, FICZ induced expression of *CYP1A1* mRNA in the in vitro skewed Th17 cells and, to a lesser extent, in bulk CD4+ T cells (Supplementary Figure S3a). Importantly, CYP1A1 enzymatic activity induced by FICZ was significantly higher in skewed Th17 cells (a 7.3-fold increase over vehicle control) than in bulk CD4+ T cells (a 3.8-fold increase over vehicle control) (Figure 5c). To further confirm that CYP1A1 activity within our culture was restricted to Th17 cells, we FACS sorted the skewed Th17 cells stimulated with either FICZ or vehicle control into CCR6+ and CCR6− cells (Supplementary Figure S3b) and found that only CCR6+ cells displayed CYP1A1 activity in response to FICZ (Supplementary Figure S3c), highlighting the importance to account for possible variability in the proportions of in vitro skewed Th17 cells in each culture. Therefore, when comparing patients with psoriasis (n = 15) and healthy controls (n = 20), we measured the amount of IL-17A in the supernatant of each culture and subsequently used it to normalize CYP1A1 activity. We found that cells of patients with psoriasis had significantly higher CYP1A1 activity than those of healthy controls (Figure 5d). Because our clinical samples were not sex matched, we also compared CYP1A1 activity in males and females but found no significant difference in either the overall cohort (Supplementary Figure S3d) or in patients with psoriasis or healthy controls stratified by sex (Supplementary Figure S2e and f). We also controlled for the smoking status of the participants because cigarette smoke contains PAH-activating AHR (Stockinger et al., 2014). CYP1A1 activity remained significantly increased in patients with psoriasis compared with that in healthy controls when the analysis was restricted to nonsmoker individuals (Supplementary Figure S3g). Finally, because three of the patients included in our study were to commence biological treatment with adalimumab, we measured their CYP1A1 activity again at week 12 and found no significant difference compared with their activity before treatment, suggesting that CYP1A1 activity is not modified by biological treatment (Supplementary Figure S2h).

## Discussion

The AHR pathway is increasingly recognized as a critical regulator of tissue homeostasis (Rothhammer and Quintana, 2019; Stockinger et al., 2014), particularly in a barrier organ such as the skin, where we and others have shown a beneficial effect of physiological AHR activation in mouse models and human clinical samples (Di Meglio et al., 2014a; van den Bogaard et al., 2013).

In this study, we identify CYP1A1 enzymatic activity as a critical downregulator of beneficial AHR signaling in skin inflammation. Mice constitutively overexpressing *Cyp1a1* displayed increased CYP1A1 enzymatic activity in the skin, which resulted in exacerbated immune cell activation and skin pathology, mirroring that observed in *Ahr*-deficient mice. Inhibition of CYP1A1 enzymatic activity ameliorated skin immunopathology. Interestingly, in agreement with our data in mice, patients with psoriasis displayed increased CYP1A1 enzymatic activity compared with healthy donors, suggesting that dysregulation of the AHR/CYP1A1 axis may play a role in inflammatory skin disease.

Activation of the AHR pathway has been considered an unpopular target for potential novel drugs for several decades owing to the well-known toxicity associated with xenobiotic exposure. The current view is that xenobiotic-dependent AHR functions represent an adaptive mechanism overlapping its otherwise beneficial role in cell physiology and organ homeostasis (Esser et al., 2018; Mulero-Navarro and Fernandez-Salguero, 2016). Moreover, it is increasingly clear that the overall consequences of AHR activation in vivo depend on the duration of the signal (Mitchell and Elferink, 2009) and the tissue context (Haarmann-Stemmann et al., 2015).

Prolonged AHR signaling induced by nondegradable xenobiotics, such as dioxin, and bioactivated compounds, such as benzo[α]pyrene-derivatives, results in unwarranted immunosuppression, skin pathology (e.g., chloracne), and carcinogenesis (Mitchell and Elferink, 2009). On the contrary, transient activation mediated by nontoxic physiological ligands constraints inflammation and contributes to epidermal barrier formation (Di Meglio et al., 2014a; van den Bogaard et al., 2015). In keeping with this dichotomy, mice expressing a constitutively active form of AHR spontaneously developed skin features resembling atopic inflammation, similar to that induced by the PAH 7,12-dimethylbenz[a]anthracene in wild-type mice (Hidaka et al., 2017). Conversely, prolonged exposure to FICZ did not exert any toxic effects in the same study (Hidaka et al., 2017) and was beneficial in others (Di Meglio et al., 2014a; Smith et al., 2017). Finally, because AHR activation may have detrimental consequences in healthy tissue, for example, inducing photocarcinogenesis in mice (Pollet et al., 2018), the choice between activation and inhibition has to be carefully considered (Haarmann-Stemmann et al., 2015).

Central to the multifaceted effect of AHR activation is CYP1A1, which catalyzes the bioactivation of PAH to oxygenated reactive intermediates capable of reacting with DNA and initiating mutagenesis and carcinogenesis (Santes-Palacios et al., 2016). Nevertheless, CYP1A1 detoxifies PAHs and other xenobiotics into polar derivatives excreted in the urine and bile (Santes-Palacios et al., 2016). Importantly, murine studies of oral ingestion of benzo[α]pyrene have shown that the beneficial effects of CYP1A1-mediated detoxification are far more prominent than the detrimental effects mediated by xenobiotics bioactivation (Nebert et al., 2013). Moreover, CYP1A1 rapidly degrades physiological AHR ligands, such as FICZ (Effner et al., 2017; Wincent et al., 2009), thus regulating the duration of AHR signaling and preventing severe immunosuppression. Moreover, the dysregulated expression of *Cyp1a1* depletes the reservoir of natural AHR ligands in *R26*^*Cyp1a1*^ mice and has a negative impact on intestinal immunity (Schiering et al., 2017).

In this study, we show that constitutive expression of *Cyp1a1* resulted in increased CYP1A1 enzymatic activity in the skin of *R26*^*Cyp1a1*^ mice, in the absence of any overt skin alteration at the cellular level, with the noticeable exception of epidermal γδ T cells, which were drastically reduced, as seen in *Ahr*-deficient mice (Li et al., 2011). Survival of epidermal γδ T cells in the skin depends on AHR signaling, and their dramatic reduction in the skin of *R26*^*Cyp1a1*^ mice is a direct consequence of the likely AHR ligands’ depletion occurring in the presence of increased CYP1A1 enzymatic activity. Although this cell subset does not exist in humans, epidermal γδ T cells play a key role in wound healing in mice, and their dependency on AHR signaling for survival confirms its homeostatic role in the skin.

The effect of increased CYP1A1 metabolic activity and potential reduction of AHR ligands was much more prominent in *R26*^*Cyp1a1*^ mice undergoing psoriasis-like inflammation. AHR signaling limits inflammation by damping proinflammatory responses in KCs, particularly the production of chemokines and cytokines recruiting and further activating immune cells, such as IL-17–producing cells into the site of inflammation (Di Meglio et al., 2014a). Increased metabolic activity in *R26*^*Cyp1a1*^ mice faithfully phenocopied *Ahr* deficiency, leading to exacerbated skin inflammation, which in turn was attenuated by the inhibition of CYP1A1 enzymatic activity by α-NF, a potent CYP1 inhibitor (Wincent et al., 2012), also described in early studies as a partial AHR antagonist (Merchant et al., 1993).

Interestingly, inhibition of physiological CYP1A1 activity in wild-type mice did not influence Aldara-induced skin inflammation (data not shown), suggesting that the metabolic turnover of AHR physiological ligands is carefully optimized under normal conditions, in keeping with the known harmful effects of electrophilic compounds produced during xenobiotics bioactivation.

A recent study has suggested that imiquimod, the active component of Aldara, is a substrate for CYP1A1 and CYP1A2 enzymes and that the exacerbated skin inflammation detected in the absence of AHR signaling results from the prolonged action of not-metabolized imiquimod rather than from the lack of AHR-mediated anti-inflammatory mechanisms (Mescher et al., 2019). Nevertheless, in this study, we show that CYP1A1 overexpression indeed exacerbates rather than reduces Aldara-induced inflammation, thus strengthening the notion that AHR signaling per se is beneficial to constraint inflammation.

In keeping with the beneficial effect of physiological AHR signaling, we detected activation of the AHR pathway during Aldara-induced psoriasiform skin inflammation. Increased CYP1A1 has been detected in the inflamed skin of patients with atopic dermatitis (Hidaka et al., 2017) and in the PBMCs of patients with systemic lupus erythematosus (Shinde et al., 2018), suggesting that upregulation of the pathway during inflammation and autoimmunity may occur in an attempt to restrict immunopathology. However, in agreement with the findings in multiple sclerosis (Rothhammer et al., 2016), we detected significantly reduced CYP1A1 expression in psoriasis lesional skin compared with that in nonlesional psoriasis and healthy skin, suggestive of an overall dysregulation of the AHR pathway in psoriasis where its anti-inflammatory effect may be impaired. It is also important to distinguish between CYP1A1 expression and activity, with decreased *CYP1A1* gene expression counteracted by increased CYP1A1 enzymatic activity in various scenarios.

A diverse range of physiological AHR ligands has been shown to ensure homeostatic regulation in different organs. The tryptophan photoproduct FICZ is readily present in human skin, and microbiota-derived ligands have also been described (Shinde and McGaha, 2018). Hence, there should be no shortage of AHR ligands available in the skin to maintain homeostasis. However, both intrinsic (genetic) or extrinsic (environmental) factors can influence CYP1A1 inducibility and/or enzymatic activity and thus affect ligand availability. Interestingly, circulating levels of AHR ligands are decreased in individuals with MS (Rothhammer et al., 2016), raising the possibility that reduced ligand availability underpins defective AHR signaling in autoimmune diseases.

We detected significantly increased CYP1A1 enzymatic activity in patients with psoriasis compared with that in healthy controls. In the *R26*^*Cyp1a1*^ mouse, aberrant CYP1A1 enzymatic activity phenocopies AHR deficiency with exacerbated skin pathology. A similar scenario might take place in psoriasis. Activation of the AHR pathway may take place initially, attempting to restrict inflammation, but could be rapidly abolished by accelerated ligand metabolism owing to aberrant CYP1A1 activity, thus preventing AHR anti-inflammatory effect. Studies investigating AHR ligand availability in patients with psoriasis and in healthy controls are crucially needed to verify this hypothesis.

The microbial derivative AHR ligand tapinarof has shown clinically meaningful dose-dependent improvements in phase IIb trials in both psoriasis and atopic dermatitis (Peppers et al., 2019; Robbins et al., 2019); it is currently being tested in phase III trials for psoriasis. Our study expands on the recent findings by Effner et al. (2017) where CYP1A1 inhibition resulted in altered AHR-mediated immune responses in vitro and verifies its consequences and applicability in the context of skin inflammation.

In this study, we provide evidence that therapeutic targeting of the AHR pathway can be achieved by other strategies, namely inhibition of CYP1A1 enzymatic activity to increase the availability of physiological AHR ligands. This can be of importance in the presence of increased CYP1A1 activity as we have detected in patients with psoriasis versus that in controls.

Several SNPs in the *CYP1A1* gene have been linked to increased enzymatic activity (Ingelman-Sundberg, 2004), and their contribution to the difference detected in patients with psoriasis remains to be investigated. Sex differences have also been reported, with females displaying reduced enzymatic activity (Smart and Daly, 2000). While acknowledging that our cohorts were not fully sex matched, we did not detect any difference in CYP1A1 activity when either the overall cohort or patients and healthy controls were each stratified by sex, suggesting that the differences observed were not influenced by the sex of the participants. An important extrinsic factor modulating CYP1A1 activity is tobacco smoke, which contains PAH-activating AHR (Esser and Rannug, 2015). Moreover, smoking increases psoriasis risk (Naldi, 2016). Nevertheless, CYP1A1 activity remained significantly increased in Th17 cells of patients with psoriasis compared with the activity in Th17 cells of healthy controls when our analysis was restricted to nonsmoker individuals.

Although they are the main pathogenic cells in psoriasis, Th17 cells were our choice of cells to measure CYP1A1 activity in humans because they can be easily activated to express enzymatically active CYP1A1 while at the same time requiring less invasive procedures and thus being more easily accessible than primary KCs. Nevertheless, because we are not dissecting the pathogenic role of Th17 cells in psoriasis but rather using them as a robust model system to measure CYP1A1 enzymatic activity, their usage is not in contrast with the beneficial role of the AHR pathway. Moreover, increased CYP1A1 activity in Th17 cells could potentially contribute to skin inflammation in patients with psoriasis by limiting the activation of AHR signaling in KCs. Studies are going on to dissect the potential role of Th17 cells and verify our in vitro findings in primary human tissue.

Taken together, our study shows that CYP1A1 feedback regulates skin immunity and that the inhibition of CYP1A1 activity represents an alternative strategy to harness the anti-inflammatory effect exerted by physiological activation of the AHR pathway in the skin.

## Materials and Methods

### Human subjects

A total of 32 patients with psoriasis, recruited at either Guy’s and St Thomas’ Hospital (London, United Kingdom) or at the University Medical Center Schleswig-Holstein (Kiel, Germany), were included in this study. The German cohort has been previously described (Di Meglio et al., 2014a). Patients were not receiving any conventional or biological systemic therapy at the time of sampling. A total of 62 healthy controls were recruited at the Francis Crick Institute (London, United Kingdom) or at Guy’s and St Thomas’ Hospital to donate either blood or discarded healthy skin from plastic surgery procedures. Full demographics and clinical information can be found in Supplementary Table S1. The study was conducted in accordance with the Declaration of Helsinki, with written informed consent obtained from each volunteer, and was approved by the institutional review boards of the University of Kiel Medical School (Kiel, Germany), Guy’s and St Thomas’ Hospital, and the Francis Crick Institute.

### Mice

Wild-type C57Bl/6, *AhR*^*−/−*^ (Schmidt et al., 1996), and previously described *R26*^*Cyp1a1*^ and *Cyp1a1*^*Cre*^ × *R26R*^*eYFP*^ (Schiering et al., 2017) mice were bred in the Francis Crick Institute animal facility under specified pathogen-free conditions. All mice procedures were conducted under a project license granted by the United Kingdom Home Office.

### Aldara-induced psoriasiform skin inflammation

Psoriasiform skin inflammation was induced in 8-week-old female mice by treating shaved dorsal skin with 50 mg Aldara cream containing 5% imiquimod (Meda AB, Solna, Sweden) daily for 5 consecutive days, as previously described (Di Meglio et al., 2014a). In some experiments, mice were treated intraperitoneally with FICZ (100 mg/kg in corn oil) or α-NF (5 mg/kg in corn oil) or vehicle control for 7 days, starting 2 days before Aldara application. Full-thickness skin biopsies were collected the day after the last Aldara application, and skin was either snap frozen in liquid nitrogen for RNA or protein extraction, immediately digested to obtain single-cell suspensions, or fixed for histopathology and immunofluorescence analysis (Supplementary Materials and Methods). In the α-NF experiment, tail skin was collected at different time points and used to measure CYP1A1 activity. Shaved-only mice were used for analysis under a homeostatic condition (naive mice).

### CD4+ purification and in vitro skewing to Th17 cells

CD4+ T cells were purified from thawed PBMCs (Supplementary Materials and Methods) using the EasySep negative selection kit (STEMCELL Technologies, Vancouver, British Columbia, Canada) according to the manufacturers’ instructions. Purity was over 95%. Bulk CD4+ T cells from patients with psoriasis and healthy controls were cultured with a Th17-skewing cocktail to simultaneously differentiate naive T cells into Th17 cells and expand existing memory Th17 cells. CD4+ T cells were cultured in duplicate at a density of 2 × 10^6^ cells/ml in U-bottomed polystyrene tubes in RPMI 1640 medium (Thermo Fisher Scientific, Waltham, MA) containing 1% penicillin-streptomycin and 10% heat-inactivated human serum (10% HS-RPMI). They were activated with anti-CD3 and anti-CD28‒coated beads (1:1 bead-to-cell ratio, Dynabeads CD3/CD28 T cell Expander, Thermo Fisher Scientific) with or without recombinant human IL-23 (50 ng/ml), IL-1β (50 ng/ml), and TGF-β (0.5 ng/ml) (all from Miltenyi Biotec, Bergisch Gladbach, Germany) for 72 hours at 37 ^o^C. FICZ (5 nm, ENZO, Farmingdale, NY) in DMSO or DMSO (0.005% v/v) as vehicle control were added for additional 24 hours. On day 4, cell supernatant was collected and stored at −80 ^o^C for further analysis, and cell pellets were used to measure CYP1A1 enzymatic activity.

### EROD assay

CYP1A1 enzymatic activity was measured in mouse tail epidermal sheet biopsies and in vitro skewed Th17 cells by the EROD assay, in which the 7-ethoxyresorufin (Sigma-Aldrich) is converted to resorufin (Sigma-Aldrich). Tail mouse skin was processed by the mechanical removal of subcutaneous fat tissue and floated on 0.5% w/v trypsin (Sigma-Aldrich) for 1 hour at 37 °C. Epidermal sheets were mechanically separated, and quadruplicate from each epidermal sheet was transferred to a 96-wells plate. EROD reaction was initiated by adding 2 μM 7-ethoxyresorufin in sodium phosphate buffer (50 mM, PH 8.0) to each well. The plate was incubated at 37 ^o^C for 20 minutes, the reaction was stopped by adding fluorescamine (150 μg/ml in acetonitrile), and epidermal sheets biopsies were discarded. Resorufin formation was measured against a resorufin standard curve on a plate reader (FLUOstar Omega, BMG LabTech, Ortenberg, Germany) with excitation and emission wavelengths of 535 and 590, respectively. CYP1A1 enzymatic activity was normalized for the skin protein content simultaneously determined by fluorescamine fluorescence of a standard curve of BSA (Sigma-Aldrich) at excitation and emission wavelengths of 390 and 480, respectively. In vitro skewed Th17 cells were assayed similarly, and CYP1A1 enzymatic activity was normalized to the IL-17 secreted in each well.

### Statistics

Statistical analysis was performed using Prism, version 8.0 (GraphPad Software, La Jolla, CA). For in vivo experiments, values are expressed as the mean ± SEM of the number of mice, and the data shown are representative of at least two independent experiments. Comparisons were calculated by unpaired *t*-test or one-way ANOVA and Tukey’s test for multiple comparisons, as appropriate. For experiments with human samples, data were assessed for normal Gaussian distribution with D’Agostino and Pearson omnibus normality test and then analyzed by either Mann‒Whitney test or Kruskal‒Wallis test followed by Dunn’s multiple comparison test, as appropriate. The level of statistically significant difference was defined as *P* ≤ 0.05.

### Data availability statement

No large dataset was generated or analyzed in this study.

## ORCIDs

Mariela Kyoreva: http://orcid.org/0000-0002-3670-9404

Ying Li: http://orcid.org/0000-0002-8685-6314

Mariyah Hoosenally: http://orcid.org/0000-0001-6110-6085

Jonathan Hardman-Smart: http://orcid.org/0000-0002-1653-7908

Kirsten Morrison: http://orcid.org/0000-0002-5308-0249

Isabella Tosi: http://orcid.org/0000-0002-2638-8539

Mauro Tolaini: http://orcid.org/0000-0003-1020-4245

Guillermo Barinaga: http://orcid.org/0000-0002-9021-9975

Brigitta Stockinger: http://orcid.org/0000-0001-8781-336X

Ulrich Mrowietz: http://orcid.org/0000-0002-9539-0712

Frank O. Nestle: http://orcid.org/0000-0003-1033-5309

Catherine Smith: http://orcid.org/0000-0001-9918-1144

Jonathan N. Barker: http://orcid.org/0000-0002-9030-183X

Paola Di Meglio: http://orcid.org/0000-0002-2066-7780

## Conflict of Interest

FON is presently an employer of Sanofi. The remaining authors state no conflict of interest.

## Supplementary Materials and Methods

### Skin histopathology

Skin tissue was fixed in neutral buffered formalin (Sigma-Aldrich, St. Louis, MO), embedded in paraffin, and sectioned. Tissue sections were deparaffinized and stained with H&E for histological analysis. Images were acquired at ×10 magnification with a VS120 slide scanner (Olympus, Tokyo, Japan), and mean acanthosis was quantified by a researcher blind to the experimental groups by taking five measurements per three sections for each mouse.

### Skin immunofluorescence

Skin tissue was fixed in 4% paraformaldehyde (Sigma-Aldrich) at 4 °C for 6 hours followed by incubation in 30% sucrose overnight. Tissues were embedded in optimal cutting temperature medium (VWR International, Radnor, PA) followed by cryosectioning. Slides were blocked with rabbit and rat serum and stained with conjugated anti-mouse CD45 (BioLegend, San Diego, CA), anti-mouse CD3e (BioLegend), and anti-GFP (Thermo Fisher Scientific, Waltham, MA) and visualized using a Leica Confocal SP5-Invert microscope (Leica Microsystems, Wetzlar, Germany).

### Flow cytometry analysis

Single-cell skin suspension of mouse skin was prepared as previously described (Di Meglio et al., 2014). Cells were stained for CD45, TCRβ, TCRγδ, Ly6G, and CD11b (all from BioLegend) and acquired on a Fortessa (BD Biosciences, Franklin Lakes, NJ). Intracellular cytokine staining for IL-17A and IL-22 (all from BioLegend) was performed as previously described (Di Meglio et al., 2014).

### PBMCs isolation

PBMCs were purified by centrifugation on a density gradient (STEMCELL Technologies, Vancouver, British Columbia, Canada), cryopreserved in RPMI 1640 (Thermo Fisher Scientific) and/or 11.25% human serum albumin solution (Gemini Bio-Products, West Sacramento, CA) and/or 10% DMSO (Sigma-Aldrich), and stored in liquid nitrogen until further use.

### Gene expression analysis

Total RNA was isolated from cultured cells using the RNeasy Plus Mini Kit (Qiagen, Hilden, Germany) and from human and mouse skin using a Polytron PT 3000 tissue homogenizer (Kinematica AG, Malters, Switzerland), alongside with mirVana Kit or TRIzol (both from Thermo Fisher Scientific), all according to the manufacturers’ instructions. RNA was quantified and reverse transcribed into cDNA using High Capacity cDNA Reverse Transcription Kit (Thermo Fisher Scientific). Gene expression was assessed by quantitative RT-PCR using TaqMan assays (Thermo Fisher Scientific), according to the manufacturers’ instructions. For each sample, mRNA abundance was normalized to the amount of mouse β-2-microglobulin (Thermo Fisher Scientific) or HuPO (Thermo Fisher Scientific). Data analysis was performed using the ΔCt method with results expressed as mRNA relative expression to β-2-microglobulin or HuPO ×1,000 in arbitrary units.

### Skin protein extracts

Skin tissue was homogenized in NP-40 Lysis buffer (Invitrogen, Carlsbad, CA) supplemented with cOmplete Protein Inhibitor Cocktail (Roche Holdings AG, Basel, Switzerland) using Polytron PT 3000 tissue homogenizer at maximum speed on ice. Homogenates were vortexed for 30 seconds and chilled on ice for 10 minutes and were spun down at 13000 r.p.m. at 4 ^o^C for 10 minutes, and the supernatants were aliquoted and stored at −80 ^o^C until further use.

### Cytokine measurements

Cytokine levels in cell supernatants or mouse skin protein extracts were assayed using customized Milliplex MAP Human Cytokine/Chemokine Kit or the Milliplex MAP Mouse Th17 Kit (both from Merck Millipore, Burlington, MA) and acquired on a FlexiMap three-dimensional flow-based sorting and detection analyzer (Luminex Corporation, Austin, TX).

### FACS

In vitro skewed human T helper type 17 cells were stained for CCR6 (clone 11A9, BD Biosciences) and sorted into CCR6+ and CCR6− cells to 99% purity on a BD Influx (BD Biosciences) and then immediately used to measure ethoxyresorufin-O-deethylase activity.
